# Feature-Based Molecular Networks Identification of Bioactive Metabolites from Three Plants of the Polynesian Cosmetopoeia Targeting the Dermal Papilla Cells of the Hair Cycle

**DOI:** 10.3390/molecules27010105

**Published:** 2021-12-24

**Authors:** Kristelle Hughes, Raimana Ho, Stéphane Greff, Gaëtan Herbette, Edith Filaire, Edwige Ranouille, Jean-Yves Berthon, Phila Raharivelomanana

**Affiliations:** 1 EIO UMR 241, IFREMER, ILM, IRD, Université de la Polynésie Française, BP 6570, F-98702 Faaa, Tahiti, French Polynesia; kristelle.hughes@doctorant.upf.pf (K.H.); raimana.ho@upf.pf (R.H.); 2 Institut Méditerranéen de Biodiversité et d’Ecologie Marine et Continentale (IMBE), UMR 7263 CNRS, IRD, Aix Marseille Université, Avignon Université, Station Marine d’Endoume, rue de la Batterie des Lions, 13007 Marseille, France; stephane.greff@imbe.fr; 3 Aix Marseille Université, CNRS, Centrale Marseille, FSCM, Spectropole, Service 511, Campus Saint-Jérome, 13397 Marseille, France; gaetan.herbette@univ-amu.fr; 4 Greentech SA, Biopôle Clermont-Limagne, 63360 Saint-Beauzire, France; edithfilaire@greentech.fr (E.F.); developpement@greentech.fr (E.R.); jeanyvesberthon@greentech.fr (J.-Y.B.); 5 UMR 1019 INRA-UcA, UNH (Human Nutrition Unity), ECREIN Team, Université Clermont Auvergne, 63000 Clermont-Ferrand, France

**Keywords:** cosmetopoeia, metabolomics, hair growth, *Bidens pilosa*, *Calophyllum inophyllum*, *Fagraea berteroana*

## Abstract

The term cosmetopoeia refers to the use of plants in folks’ cosmetics. The aerial parts of *Bidens pilosa* L., the leaves of *Calophyllum inophyllum* L. and the fruits of *Fagraea berteroana* A.Gray ex Benth are traditionally used in French Polynesia for hair and skin care. During the hair cycle, dermal papilla cells and their interaction with epithelial cells are essential to promote hair follicle elongation. The aim of our investigations was the identification of metabolites from these three plants and chemical families responsible for their hair growth activity. A bioactivity-based molecular network was produced by mapping the correlation between features obtained from LC-MS/MS data and dermal papilla cell proliferation, using the Pearson correlation coefficient. The analyses pointed out glycosylated flavonols and phenolic acids from *B. pilosa* and *C. inophyllum*, along with C-flavonoids, iridoids and secoiridoids from *F. berteroana*, as potential bioactive molecules involved in the proliferation of hair follicle dermal papilla cells. Our results highlight the metabolites of the plant species potentially involved in the induction of hair follicle growth and support the traditional uses of these plants in hair care.

## 1. Introduction

The current natural products bioassay-guided fractioning from plant material extraction to isolation of active compounds presents several drawbacks. Indeed, finding novel molecules that also possess significant bioactivities can be time consuming and yet yield little reward (too little quantity of isolated product or in complex mixture, lack of activity in single compounds tests, degradation of sample during multiple fractioning steps). This also leads to a redundancy in isolated products as easily purifiable compounds often correspond to well-known molecules. In this regard, the development of high-resolution mass spectrometry has enabled dereplication analyses to rapidly identify known compounds or substances from available libraries [1]. Furthermore, the formerly restricted number of detectable compounds with traditional methods is lifted by untargeted molecular analyses. Molecular networks applied to natural products research have made it possible to map the true complexity and metabolite richness of plant extracts [2,3].

*Bidens pilosa* L., *Calophyllum inophyllum* L. and *Fagraea berteroana* A.Gray ex Benth are respectively a herb and two trees of the Polynesian cosmetopoeia. Their plant parts have been traditionally used as topical preparations for skin embellishment and wound healing in French Polynesia, for centuries [4,5]. All three plants showed promise in being developed as hair care ingredients according to their traditional use and available literature, based on our previous screening and review [6]. The aerial parts of *B. pilosa*, the leaves of *C. inophyllum* and the fruits of *F. berteroana* were thus previously extracted using solvents of gradual polarity (ethyl acetate, ethanol: water (50:50) and water). Ultimately, the ethyl acetate extracts of all three plants and their respective plant part showed greater bioactivity upon further investigation of their in vitro anti-inflammatory and antioxidant activity, as well as their dermal papilla cell proliferation activity and RT-qPCR regulation of several genes involved in the hair cycle [7,8]. After these bioassays, first we proceeded to LC-MS/MS analyses and were able to annotate 19 compounds but had not isolated or elucidated any using NMR, nor had the hair growth related biological activities of the compounds been assessed.

Feature-Based Molecular Networking (FBMN) provided an ideal option in such a case as it combines feature detection alignments of LC or GC-MS data with classical molecular networking to allow quantitative analysis and mapping of detected features [9,10]. Furthermore, Bioactivity-Based Molecular Networking integrates values of biological activities to the molecular network to highlight potential bioactive clusters and specific metabolites [11,12]. The bioactivity selected for this study was hair follicle dermal papilla cells (DPCs) proliferation. These cells are paramount during the anagen (or follicle elongation) phase of the hair cycle. During the elongation phase of the hair follicle, the Wnt canonical pathway is activated in DPCs, resulting in an accumulation of cytoplasmic β-catenin protein. It subsequently leads to the translocation of this β-catenin protein to the nucleus to aid in the transcription of downstream targets that interact with keratinocytes to induce follicle growth [13]. When the Wnt pathway is disturbed in balding DPCs, it stunts keratinocyte growth responsible for hair keratin production [14]. Furthermore, the number of DPCs has an impact on hair size and a decrease in their pool leads to gradual hair thinning [15].

The aim of this study was dual: firstly, to further our investigations of the chemical composition of our three plant species *B. pilosa*, *C. inophyllum* and *F. berteroana*, and secondly to highlight which metabolites or chemical families could be involved in the observed hair growth-related biological activities of the fractions/extracts obtained from the three plants. To do so, we adapted the Bioactivity-Based Molecular Networking workflow proposed by Nothias et al., (2018) [11] to study features responsible for the hair follicle dermal papilla cells proliferation activity. The raw spectral data obtained from the LC-MS/MS analysis were pre-processed in MZmine2 and quantitatively aligned to compute a feature-based molecular network. The FBMN obtained was used to determine potential bioactive compounds or clusters. A multivariate analysis, PCA was performed to identify whether the extracts had similar chemical compositions and the Pearson correlation coefficients were calculated to identify compound-activity relationship within our samples.

## 2. Results and Discussion

### 2.1. Interspecies Correlations between the Cell Proliferative Activity of the Fractions/Extracts and Their Chemical Constituents

Three plants, *B. pilosa*, *C. inophyllum* and *F. berteroana* were studied. The LC-MS/MS data from the ethyl acetate extract of the aerial parts of *B. pilosa* (BEAE) which yielded BF3 and BF4 after fractionation, the ethanol/water (CEWE) extract of *C. inophyllum* leaves, the ethyl acetate (CEAE) leaf extract of *C. inophyllum* which yielded CF3, CF4 and CF5 after fractionation and the ethyl acetate (FEAE) extract of *F. berteroana* which yielded fractions FF1, FF2, FF3 and FF4, were used for spectral alignment in MZMine.

The feature quantification table obtained from MZMine containing 1110 aligned features of the 13 fractions and extracts as well as their respective cell proliferation values, were necessary for spectral data vs biological activity correlation calculations. The mean cell proliferation values at 50 μg·mL^−1^ of the fractions/extracts were first ln-transformed (Table S1), before correlation was calculated. The output results showed that 53 of the 1110 initial features had a significant correlation with the bioactivity (*p*-value ≤ 0.05; Table S2).

The Pearson correlation coefficients obtained were mostly negative, ranging from −0.79 to −0.55. This reveals that the features demonstrated an inverse quantitative relationship with cell proliferation. Conversely, two features were significantly positively correlated to the bioactivity (Table S2). The results of the Pearson test were incorporated into the molecular network to visually plot the bioactivity scores of the features.

### 2.2. The Chemical Composition of the Bioactive Fractions

Several compounds were isolated from the three plant species and identified using 1D and 2D NMR.

#### 2.2.1. Chemical Constituents of BF4 from the Aerial Parts of *Bidens pilosa*

Fraction BF4 from the ethyl acetate extract of *B. pilosa* aerial parts was fractionated on a Luna C18 (150 mm × 10 mm, 5 μm, Phenomenex, Torrance, CA, USA) column in isocratic mode at 47% acetonitrile for 20 min using a Kontron HPLC system and gradient system from 47% acetonitrile to yield compounds 1 (0.5 mg), 2 (1 mg), 3 (0.5 mg), 4 (1.7 mg) at 11.8, 14.4, 14.7, 15.3 min of analysis, respectively.

**Figure 1:**
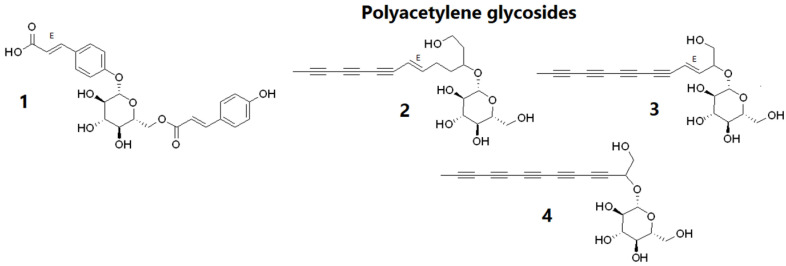
Chemical structure of compounds, 2-Propenoic acid, 3-[4-[[6-O-[(2E)-3-(4-hydroxyphenyl)-1-oxo-2-propenyl]-β-d-glucopyranosyl]oxy]phenyl] (**1**), β-d-Glucopyranoside, (4E)-1-(2-hydroxyethyl)-4-dodecene-6,8,10-triynyl (**2**), β-d-Glucopyranoside, (2E)-1-(hydroxymethyl)-2-dodecene-4,6,8,10-tetraynyl (**3**), β-d-Glucopyranoside, 1-(hydroxymethyl)-2,4,6,8,10-dodecapentaynyl (**4**) isolated from BF4 of *Bidens pilosa*.

The chemical composition of *B. pilosa* has been extensively studied because of its many biological activities. Over 200 compounds have been isolated from its plant parts, belonging to several compound families [16]. In this study, four compounds were isolated from the aerial parts of *B. pilosa* (Figure 1). One glycosylated phenylpropanoid derivative and three glycosylated polyacetylenes were isolated from BF4, and their structures were elucidated by NMR analysis as 2-Propenoic acid, 3-[4-[[6-O-[(2E)-3-(4-hydroxyphenyl)-1-oxo-2-propenyl]-β-d-glucopyranosyl]oxy]phenyl] (**1**), β-d-Glucopyranoside, (4E)-1-(2-hydroxyethyl)-4-dodecene-6,8,10-triynyl (**2**), β-d-Glucopyranoside, (2E)-1-(hydroxymethyl)-2-dodecene-4,6,8,10-tetraynyl (**3**), β-d-Glucopyranoside, 1-(hydroxymethyl)-2,4,6,8,10-dodecapentaynyl (**4**). The sugar moiety corresponded to hexose in all four cases (Figure 1). All the compounds described here have previously been isolated from *B. pilosa* [16]. While these glycosylated forms have never been tested for their hair growth activity, simple polyacetylenes such as dihydropanaxacol have shown mild inhibition of the binding of brain-derived neurotrophic factor (BDNF) to its receptor TrkB and strong inhibition of β-nerve growth factor (BNGF) and p75 neurotrophin receptor (p75NTR) binding. These neutrophins have been found to intervene in the anagen to catagen transition of the hair cycle by upregulation of TGFβ. Thus, the inhibitory activity of dihydropanaxacol prolonged the anagen, hair follicle elongation, phase [17].

#### 2.2.2. Chemical Constituents of CF5 from the Leaves of *Calophyllum inophyllum*

The CF5 fraction obtained from the ethyl acetate leaf extract of *C. inophyllum* was purified on a semi-preparative Kontron Instruments HPLC System with an ODS7 column (250 mm × 10 mm, 7 μm, Develosil, San Diego, CA, USA) at 45% acetonitrile/55% water in isocratic mode. Compound **5** eluted after 12 min (28.5 mg) and compound **6** was obtained after 17 min (1.5 mg).

**Figure 2:**
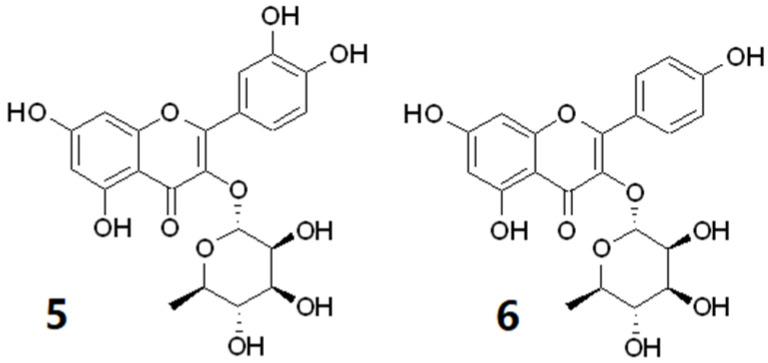
Chemical structure of compounds, quecitrin (**5**) and afzelin (**6**) isolated from CF5 of *Calophyllum inophyllum*.

Two flavonoids, quercitrin (**5**) and afzelin (**6**) were isolated and identified from the leaves of *C. inophyllum* in our study (Figure 2). Both compounds have already been observed in the leaves and stem of this species [18]. Furthermore, quercitrin has been isolated from the stems and leaves [18] and flowers of *C. inophyllum* [19] as well as the leaves of *C. incrassatum* [20] and *C. flavoramulum* [21]. Flavonoids, coumarins and triterpenoids are the main constituents reported in *C. inophyllum* leaves [22,23,24].

#### 2.2.3. Chemical Constituents of FF1 from the Fruits of *Fagraea berteroana*

Fraction FF1 from the ethyl acetate extract of the fruits of *F. berteroana* was purified on the Kontron HPLC system with a Luna C18 column (150 mm × 10 mm, 5 μm, Phenomenex, Torrance, CA, USA) in a gradient system from 70% acetonitrile to 100% acetonitrile in 20 min. Compound 7 was afforded at a retention time of 5 min (3.5 mg), compounds 8 and 9 were obtained as mixtures at 15.4 min and 16.2 min. Compounds 10 and 11 were obtained as a mixture with 4 and 5 after 16 min of analysis.

**Figure 3:**
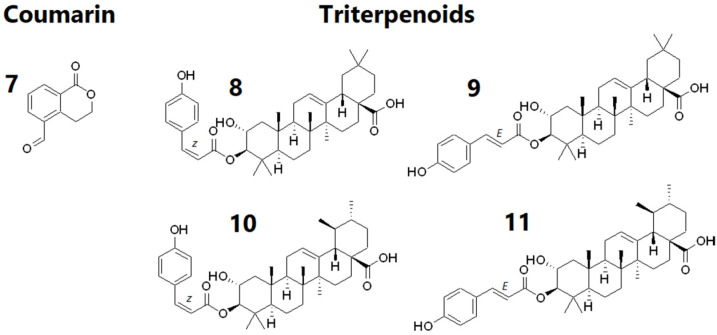
Chemical structure of compounds, erythrocentaurin (**7**), *cis*-*p*-coumaroyloxy maslinic acid (**8**), *trans*-*p*-coumaroyloxy maslinic acid (**9**), *cis*-*p*-coumaroyloxy corosolic acid (**10**) and *trans*-*p*-coumaroyloxy corosolic acid (**11**) isolated from FF1 of *Fagraea berteroana*.

Five compounds were isolated and identified from FF1 (Figure 3), a coumarin, erythrocentaurin (**7**), as well as four isomeric triterpenoids, cis-p-coumaroyloxy maslinic acid (**8**), trans-p-coumaroyloxy maslinic acid (**9**), cis-p-coumaroyloxy corosolic acid (**10**) and trans-p-coumaroyloxy corosolic acid or jacoumaric acid (**11**). The cis and trans isomers p-coumaroyloxy corosolic and maslinic acids were isolated as mixtures eluting closely together as they share the same molecular formula and only differ in the position of a methyl group.

This is the first time erythrocentaurin has been isolated from a *Fagraea* species, although it has already been isolated in the Gentianaceae family [25,26].

Corosolic acid was primarily isolated from the leaves of ‘loquat’, *Eriobotrya japonica* [27] and also of ‘banaba’ *Lagerstroemia speciosa*. Both plants are used in Chinese folk medicine, including South-East Asia for banaba, as tea preparations to treat diabetes [28,29,30]. Patients unknowingly suffering from type 2 diabetes have reported diffuse hair loss and the latter could be a warning sign for the occurrence of this disease [31]. Moreover, a study on a cohort of African American women revealed an increased risk of developing severe central hair loss with type 2 diabetes [32]. One of the reasons for this association is hypothesized to be the decrease of proper blood flow [31]. Thus, loquat leaf extracts were privately studied for their hair growth activity. As a result, corosolic acid is cited as one of the active ingredients in a patented hair growth product for its proliferative activity on dermal papilla cells as well as keratinocytes [33,34]. Hence, corosolic acid is likely to be responsible for the dermal papilla cell proliferation observed in FF1 and could contribute to increased β-catenin levels of the fraction compared to the control, and *in fine*, hair growth. Maslinic acid is a triterpene that had firstly been isolated in olive pomace. It is believed to confer a waxy texture that protects against plant pathogens. Interestingly, the fruits of *F. berteroana* have a waxy coat that make them very sticky. The composition of this wax had never been studied but the similarity with the structure described in olives suggests that maslinic acid could be a component of the wax-like structure that coats the fruits of *F. berteroana*. Furthermore, a plethora of biological activities are associated with maslinic acid namely, antioxidant, antidiabetic, and anti-inflammatory by regulating lipopolysaccharides [35,36,37].

### 2.3. Bioactivity-Based Molecular Networking on Hair-Related Targets: Hair Follicle Dermal Papilla Cells Proliferation

**Figure 4:**
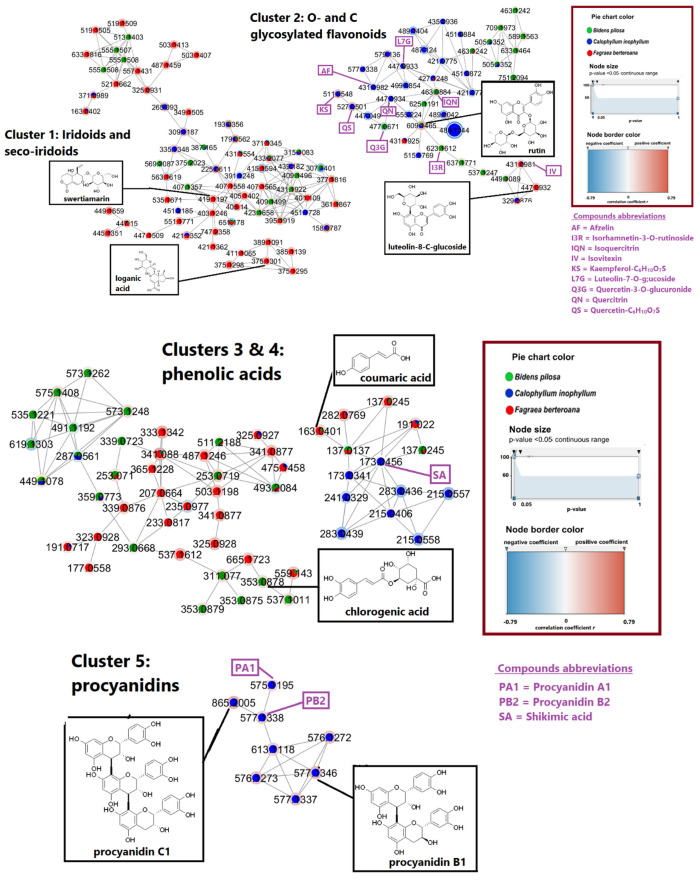
Global bioactivity-based molecular network for the three plant species *Bidens pilosa* (green), *Calophyllum inophyllum* (blue) and *Fagraea berteroana* (red). Blue node border = negative correlation and Red node border = positive correlation.

The overall interspecies cell proliferation-based molecular network in Figure 4 displayed five main clusters. Positive correlations to cell proliferation were interpreted as nodes representing features that increase cell proliferation and were observed by the light to dark red borders of the nodes. Triterpenoids and polyacetylenes isolated from *B. pilosa* and *F. berteroana* were not identified in the molecular network probably because the chromatographic or spectrometric methods used did not permit their detection. Analysis of isolated triterpenoids in mass spectrometry also demonstrated the neutral loss of the phenolic with a weak fragmentation of the terpene moiety (whatever the employed collision energy).

Cluster 1 grouped features from all three species (pie charts containing green, blue and red portions), although specific nodes were observed for *F. berteroana* within the cluster (fully red pie charts) while *B. pilosa* and *F. berteroana* shared several similar features (green and red pie charts). The annotated features were iridoids such as loganic acid and isomers *m*/*z* 375.1301 [M − H]^−^, as well as seco-iridoid swertiamarin *m*/*z* 419.1197 [M + HCOO]^−^ from *F. berteroana* extracts and fractions. This is the first time these specific compounds are observed in *F. berteroana*, although boonein, also an iridoid, was found in the fruits [8] and swertiamarin was previously isolated from *Fagraea fragrans* [38]. Furthermore, several iridoids and iridoid glycosides have been isolated from the *Fagraea* genus [38,39,40,41,42,43]. Although non-significant, most features in this cluster had positive correlations to cell proliferation as observed by the pink/red node borders. This observation suggested that iridoids, may also contribute to the proliferation of dermal papilla cells. Swertiamarin is found in a tonic that has shown a significant increase in hair density for patients suffering from premature alopecia, although the hair regrowth was mainly attributed to oleanolic acid [44]. 

Cluster 2 contained O- and C-glycosylated flavonols. As seen from the color of the pie charts, these compounds were mostly found in *C. inophyllum* and *B. pilosa*. Moreover, both afzelin and quercitrin, isolated from the CF5 fraction were successfully identified in the molecular network. Nevertheless, they both showed a negative correlation to the bioactivity as observed by blue node borders. However, isoquercitrin *m*/*z* 463.0884 [M − H]^−^ annotated from *B. pilosa* and rutin *m*/*z* 609.1465 [M − H]^−^ found in all three species, both O-glycosylated flavonoids as well, were positively correlated.

Two positively correlated C-glycoside flavonoids were annotated, one exclusively found in a *F. berteroana* fraction corresponding to luteolin-8-C-glucoside *m*/*z* 447.0932 [M − H]^−^ and one found both in *C. inophyllum* and *F. berteroana*, isovitexin m/z 431.0981 [M − H]^−^. C-glycosylated flavonols have never been isolated and observed in the leaves of *C. inophyllum*, nor *F. berteroana* extracts previously. Nonetheless, taxifolin-6-C-glucoside and aromadendrin-6-C-glucoside were discovered in the stem barks of *Fagraea auriculata* and *Fagraea ceilanica* respectively [43]. Furthermore, kaempferol-C_6_H_10_O_7_S *m*/*z* 511.0548 [M − H]^−^ and quercetin-C_6_H_10_O_7_S *m*/*z* 527.0501 [M − H]^−^, sulfur containing metabolites previously detected in *C. inophyllum* [7], showed a positive correlation to the cell proliferation.

All in all, while known compounds such as quercitrin and closely related O-glycosylated flavonols present in *C. inophyllum* were negatively correlated to cell proliferation (some significantly), C-glycosylated flavonols present in *F. berteroana* and *C. inophyllum* showed positive correlation patterns, although non-significant. These findings are supported by literature as flavonoids and flavonoid glycosides have been reported to have an inducing effect on hair growth and thus hair length, as well as hair width including a protective effect [45,46].

Clusters 3 & 4 contained phenolic acids such as chlorogenic acid (cluster 3) *m*/*z* 353.0878 [M − H]^−^ and p-coumaric acid (cluster 4) *m*/*z* 163.0401 [M − H]^−^. The latter was previously identified in our extracts [7]. While cluster 3 was mainly composed of features present in *B. pilosa* and *F. berteroana* fractions, cluster 4 grouped features mostly present in *C. inophyllum*. Furthermore, the mass formulae of the features in cluster 3 suggested the presence of a class of flavonoids potentially different from those of cluster 2. Cluster 4 likely corresponded to simple phenolic acids (smaller masses observed) such as shikimic acid *m*/*z* 173.0341 [M − H]^−^.

Features in cluster 5 stood out as only being present in *C. inophyllum* (blue pie charts) while having a positive correlation to cell proliferation, though not significantly. These corresponded to procyanidins: procyanidin A1 *m*/*z* 575.1195 [M − H]^−^, B1 *m*/*z* 577.1346 [M − H]^−^ and B2 *m*/*z* 577.1338 [M − H]^−^ and procyanidin C1 *m*/*z* 865.2005 [M − H]^−^. This result is interesting as procyanidin B2 has already been studied for hair growth activity and has shown a down-regulation of PKC isozymes, resulting in the proliferation of epithelial cells [47] needed for hair follicle elongation.

The observed correlation trends provided insight into the metabolites involved in regulating the studied bioactivity. A specific class of glycosylated flavonoids potentially intervenes in promoting cell proliferation in all three plant species, while iridoids may also account for the proliferation of dermal papilla cells after treatment by *F. berteroana* extracts and fractions. Lastly, procyanidins A-C are likely bioactive compounds from *C. inophyllum* that have several mechanistic targets including dermal papilla cell proliferation. The activity of the several classes of compounds could be mediated via the Wnt pathway, and thus potentially target the upregulation of β-catenin levels. The full list of annotated metabolites from the five clusters as well as the two significantly positively correlated metabolites from the R analysis are presented in Table 1.

### 2.4. Multivariate Analysis of Interspecies Mapping with Principal Component Analysis

**Figure 5:**
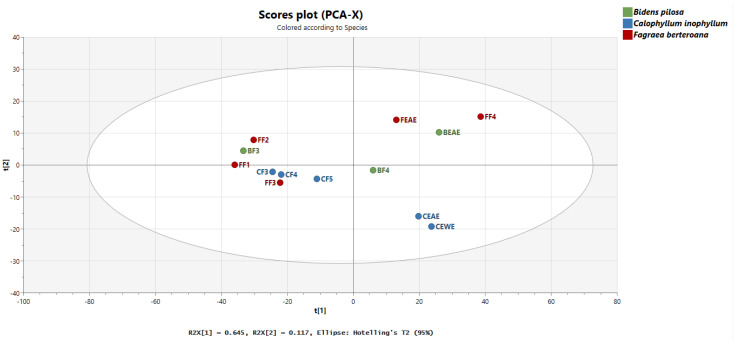
Principal Component Analysis of the three plant species. BEAE: *Bidens pilosa* Ethyl Acetate Extract and BF3, BF4 fractions, CEWE: *Calophyllum inophyllum* Ethanol-Water Extract, CEAE: *Calophyllum inophyllum* Ethyl Acetate Extract and CF3, CF4, CF5 fractions, FEAE: *Fagraea berteroana* Ethyl Acetate Extract and fractions FF1, FF2, FF3, FF4.

The extracts and fractions were first analyzed by PCA to observe global trends according to species in Figure 5. These consisted of the ethyl acetate extract of *B. pilosa* (BEAE) and its most polar fractions BF3 and BF4, the ethanol/water (CEWE) and ethyl acetate (CEAE) extracts of *C. inophyllum* with fractions CF3, CF4 and CF5, and finally the ethyl acetate (FEAE) extracts of *F. berteroana* with fractions of increasing polarity FF1, FF2, FF3 and FF4.

According to the scores plot in Figure 5, the first component explained 65% of the variance while the second component explained 12% of the variance. Indeed, most fractions were grouped in the left part of the plot, while extracts were grouped to the right. The most polar fraction of *B. pilosa*, BF4 and the most polar fraction of *F. berteroana*, FF4 were also observed on the right side of the plot. Along the second component, BEAE/FEAE/FF4 and CEAE/CEWE showed distinct groupings from fractions BF3, CF3, CF4, CF5, FF1, FF2 and FF3. This lack of distinctive grouping amongst fractions shows that there are many metabolites that are common to the bioactive fractions. This is also observable in the molecular network in which metabolites are present in several species. Nonetheless, the distinct grouping of the extracts shows that at the extract level, the chemical composition varies more. The plotting also suggests that the chemical composition of *F. berteroana* and *B. pilosa* have a higher percentage of similarity amongst themselves compared to *C. inophyllum*. According to the molecular network in Figure 4, these common metabolites could correspond to cluster 3, phenolic acids that is mainly represented by these two species as well as cluster 1 with several iridoids and seco-iridoids in common. In contrast, cluster 5 is exclusively represented in *C. inophyllum* and clusters 2 and 4, demonstrate areas with metabolites solely from this plant species.

## 3. Materials and Methods

### 3.1. Plant Material

Three plants were collected in Tahiti, French Polynesia between December 2017, and April 2018. The aerial parts of *Bidens pilosa* L., the leaves of *Calophyllum inophyllum* L. and the fruits of *Fagraea berteroana* A.Gray ex Benth. were thus identified by botanist Dr. Butaud and vouchers [JF BUTAUD & K HUGHES 3594; K HUGHES 8 and K HUGHES 4] were deposited at the herbarium of French Polynesia (PAP).

### 3.2. Plant Extraction and Compound Isolation

The plant parts were dried in an oven at 40 °C during 48 h and the dried material was ground to a fine powder. The obtained powders of the three plants were macerated in ethyl acetate (EA) during 12 h, under agitation. An extra ethanol:water (50:50; EW) extraction was performed specifically for *C. inophyllum* in the same conditions. Ethyl acetate was removed by evaporation until a solid extract was obtained. After thin layer chromatography (TLC) analysis of resulting extracts, *B. pilosa* ethyl acetate extracts (BEAE) were fractionated using the Combiflash Companion Teledyne Isco (Teledyne ISCO, Lincoln, Dearborn, MI, USA) along with a 220 g RediSep Rf Teledyne Isco column (Teledyne ISCO, Lincoln, USA) in normal phase with silica gel. The flow rate was set at 100 mL·min^−1^ with ethyl acetate (A) and cyclohexane (B). The elution program consisted of a gradient solvent system from 80% to 50% solvent B for 5 min, followed by 8 min of 50% B in isocratic mode, then 8 min of 30% B. This was followed by 8 min of 20% B, then 15 min of 100% A and ended with 10 min in 100% methanol. The same method was applied to *C. inophyllum* ethyl acetate extract (CEAE) and generated approximately 200 fraction tubes each for BEAE and CEAE. The subfractions of each plant extract were combined through TLC and HPLC analysis and controlled to obtain main fractions of increasing polarity.

A total mass of 10 g of BEAE yielded four main fractions: BF1 (1.8 g), BF2 (2.9 g), BF3 (0.5 g), and BF4 (3.1 g).

A total mass of 10 g of CEAE yielded five main fractions: CF1 (1.3 g), CF2 (3.5 g), CF3 (0.8 g), CF4 (1.5 g) and CF5 (0.5 g). CEAE was later re-fractioned on the Combiflash system to obtain more of CF5. The HPLC-UV chromatogram of the new CF5 was controlled to ensure an identical profile to the previous fraction.

The *Fagraea berteroana* ethyl acetate extract (FEAE) was fractioned (10.9 g) using open column chromatography with silica gel 60 Å. The solvents used for gradient elution were cyclohexane, ethyl acetate and methanol in a gradually polar mixture of solvents. The elution started with two column volumes of cyclohexane/EtOAc (80/20) followed by one column volume of cyclohexane/EtOAc (60/40) which led to the first fraction, FF1 (3.9 g). The second fraction, FF2 (4.6 g), was obtained by recovering and evaporating the 2nd volume of cyclohexane/EtOAc (60/40) and the first volume of cyclohexane/EtOAc (70/30). FF3 (0.1 g) was composed of the 2nd volume of cyclohexane/EtOAc (70/30) and 100% EtOAc, and finally FF4 (2.3 g) was the fraction eluted with 100% methanol.

FEAE was also re-fractioned in open column chromatography to obtain a greater quantity of FF1.

1D and 2D NMR spectra were recorded with a Bruker Avance II+ spectrophotometer at 600 MHz coupled with a Cryoprobe TCI operating under TopSpin 3.2.PL5. Data were processed on TopSpin 4.1.1.

2-Propenoic acid, 3-[4-[[6-O-[(2E)-3-(4-hydroxyphenyl)-1-oxo-2-propenyl]-β-d-glucopyranosyl]oxy]phenyl] (**1**) ^1^H-NMR (DMSO-*d*6, 600 MHz) δ in ppm 7.52 (1H, d, J = 15.8), 7.46 (2H, d, *J* = 8.4), 7.33 (2H, d, *J* = 8.6), 7.13 (1H, d, *J* = 15.7), 6.98 (2H, d, *J* = 8.6), 6.82 (2H, d, *J* = 8.4), 6.29 (1H, d, *J* = 15.8), 6.20 (1H, d, *J* = 15.7), 4.91 (1H, d, *J* = 7.6), 4.47 (1H, dd, *J* = 12.0, 1.8), 4.12 (1H, dd, *J* = 12.0, 7.8), 3.67 (1H, ddd, *J* = 10.0, 7.8, 1.8), 3.34 (1H, brt, *J* = 8.8), 3.28 (1H, dd, *J* = 9.0, 7.6) and 3.20 (1H, brt, *J* = 9.2). ^13^C-NMR (DMSO-*d*6, 150 MHz data from HSQC and HMBC) δ in ppm 171.6, 166.6, 157.4, 145.4, 145.2, 136.8, 130.3, 130.0, 128.3, 126.3, 123.5, 117.0, 116.5, 112.8, 100.0, 76.5, 73.9, 73.2, 70.4 and 63.4. HRMS *m/z* 471.1298 [M − H]^−^ (calcd. for C_24_H_23_O_10_^−^, 471.1297). Data were consistent with published values [48].

β-d-Glucopyranoside, (4E)-1-(2-hydroxyethyl)-4-dodecene-6,8,10-triynyl (**2**) ^1^H-NMR (DMSO-*d*6, 600 MHz) δ in ppm 6.52 (1H, dt, *J* = 16.0, 7.1), 5.71 (1H, brd, *J* = 16.0), 4.12 (1H, d, *J* = 7.9), 3.68 (1H, m), 3.66 (1H, dd, *J* = 11.5, 2.2), 3.56 (1H, m), 3.42 (1H, m), 3.38 (1H, dd, *J* = 11.5, 6.9), 3.14 (1H, brt, *J* = 8.8), 3.08 (1H, ddd, *J* = 9.6, 6.9, 2.2), 2.99 (1H, dd, *J* = 9.6, 8.9), 2.92 (1H, dd, *J* = 8.6, 7.9), 2.30 (2H, m), 2.03 (3H, s), 1.58 (1H, m), 1.55 (1H, m) and 1.53 (2H, m). ^13^C-NMR (DMSO-*d*6, 150 MHz partial data from HSQC and HMBC) δ in ppm 152.8, 107.5, 102.8, 80.5, 77.0, 76.7, 75.9, 75.4, 73.9, 72.7, 70.6, 66.7, 64.0, 61.7, 59.0, 57.4, 38.2, 33.4, 28.6 and 4.2. HRMS *m*/*z* 423.1660 [M + HCOO]^−^ (calcd. for C_21_H_27_O_9_, 423.1661). Data were consistent with published values [49].

β-d-Glucopyranoside, (2E)-1-(hydroxymethyl)-2-dodecene-4,6,8,10-tetraynyl (**3**) ^1^H-NMR (DMSO-*d*6, 600 MHz) δ in ppm 6.57 (1H, dd, *J* = 16.1, 5.0), 6.17 (1H, dd, *J* =16.1, 1.6), 4.33 (1H, brqd, *J* = 5.4, 1.6), 4.11 (1H, d, *J* = 7.9), 3.65 (1H, dd, *J* = 11.6, 1.8), 3.50 (1H, m), 3.42 (1H, dd, *J* = 11.6, 6.0), 3.39 (1H, dd, *J* = 11.1, 5.6), 3.13 (1H, m), 3.07 (1H, m), 3.03 (1H, m) and 2.99 (1H, dd, *J* = 8.8, 7.8). ^13^C-NMR (DMSO-*d*6, 150 MHz data from HSQC and HMBC) δ in ppm 150.7, 108.5, 101.5, 81.1, 78.1, 77.0, 76.9, 73.9, 73.6, 70.4, 64.3, 63.9, 61.5, 59.2 and 4.3. HRMS *m*/*z* 405.1185 [M + HCOO]^−^ (calcd. for C_20_H_21_O_9_^−^, 405.1191). Data were consistent with published values [49].

β-d-Glucopyranoside, 1-(hydroxymethyl)-2,4,6,8,10-dodecapentaynyl (**4**) ^1^H-NMR (DMSO-*d*6, 600 MHz) δ in ppm 4.72 (1H, brt, *J* = 5.6), 3.66 (1H, dd, *J* = 11.8, 2.0), 3.60 (1H, dd, *J* = 11.2, 5.5), 3.56 (1H, dd, *J* = 11.2, 5.8), 3.41 (1H, dd, *J* = 11.8, 6.3), 3.17 (1H, brt, *J* = 8.9), 3.12 (1H, ddd, *J* = 9.7, 6.3, 2.0), 3.02 (1H, brt, *J* = 9.3), 2.97 (1H, dd, *J* = 8.8, 7.8). ^13^C-NMR (DMSO-*d*6, 150 MHz partial data from HSQC and HMBC) δ in ppm 100.5, 81.2, 78.9, 77.3, 76.8, 73.4, 70.2, 70.0, 68.6, 63.7, 63.5, 61.6, 61.3, 61.2, 59.0, 4.3. HRMS *m*/*z* 357.0980 [M − H]^−^ (calcd. for C_20_H_19_O_9_^−^, 357.0980). Data were consistent with published values [49].

Quercitrin (Quercetin 3-O-α-L-rhamnopyranoside) (**5**) ^1^H-NMR (CD_3_OD, 500 MHz) δ in ppm 7.34 (1H, d, *J* = 2.1), 7.31 (1H, dd, *J*= 8.3, 2.1), 6.91 (1H, d, *J* = 8.3), 6.36 (1H, d, *J* = 2.0), 6.19 (1H, d, *J* = 2.0), 5.35 (1H, d, *J* = 1.5), 4.22 (1H, dd, *J* = 3.3, 1.5), 3.75 (1H, dd, *J* = 9.4, 3.3), 3.41 (1H, dq, *J* = 9.4, 6.1), 3.34 (1H, t, *J* = 9.4), 0.94 (3H, d, *J* = 6.1). ^13^C-NMR (CD_3_OD, 125 MHz) δ in ppm 179.6, 166.6, 163.2, 159.2, 158.6, 149.9, 146.5, 136.2, 123.0, 122.8, 116.9, 116.4, 105.7, 103.5, 100.0, 94.9, 73.2, 72.1, 72.0, 71.9 and 17.7. HRMS *m*/*z* 447.0927 [M − H]^−^ (calcd. for C_21_H_19_O_11_^−^, 447.0933). Data were consistent with published values [50].

Afzelin (Kaempferol 3-O-α-rhamnoside) (**6**) ^1^H-NMR (CD_3_OD, 600 MHz) δ in ppm 7.73 (2H, d, *J* = 8.8), 6.90 (2H, d, *J* = 8.8), 6.16 (1H, d, *J* = 2.0), 6.04 (1H, d, *J* = 2.0), 5.34 (1H, d, *J* = 1.7), 4.22 (1H, dd, *J* = 3.3, 1.7), 3.72 (1H, dd, *J* = 9.0, 3.3), 3.33 (1H, m), 3.32 (1H, m) and 0.92 (3H, d, *J* = 6.1). ^13^C-NMR (CD_3_OD, 150 MHz) δ in ppm 178.6, 165.8, 162.6, 162.1, 159.3, 158.2, 135.4, 131.8, 122.6, 116.7, 103.5, 103.3, 102.6, 96.7, 73.2, 72.2, 72.0, 71.9 and 17.7. HRMS *m*/*z* 431.0979 [M − H]^−^ (calcd. for C_21_H_19_O_10_^−^, 431.0984). Data were consistent with published values [50].

Erythrocentaurin (**7**) ^1^H-NMR (DMSO-*d*6, 600 MHz) δ in ppm 10.24 (1H, s), 8.23 (1H, dd, *J* = 7.8, 1.7), 8.17 (1H, dd, *J* = 7.8, 1.7), 7.67 (1H, t, *J* = 7.8), 4.53 (2H, t, *J* = 6.0), 3.50 (2H, t, *J* = 6.0). ^13^C-NMR (DMSO-*d*6, 150 MHz) δ in ppm 192.6, 163.8, 141.7, 136.4, 134.7, 132.6, 127.7, 126.4, 66.5 and 23.7. HRMS *m*/*z* 175.0399 [M − H]^−^ (calcd. for C_10_H_7_O_3_^−^, 175.0401). Data were consistent with published values [51].

*cis*-*p*-coumaroyloxy maslinic acid (**8**) ^1^H-NMR (DMSO-*d*6, 600 MHz) δ in ppm 7.67 (2H, d, *J* = 8.8), 6.80 (1H, d, *J* = 12.8), 6.72 (2H, d, *J* = 8.8), 5.78 (1H, d, *J* = 12.8), 5.11 (1H, brt, *J* = 3.5), 4.47 (1H, d, *J* = 9.8), 3.65 (1H, brtd, *J* = 10.6, 4.3), 2.82 (1H, m),1.85 (1H), 1.83 (2H), 1.80 (1H), 1.78 (1H), 1.61 (1H), 1.58 (1H), 1.57 (1H), 1.49 (1H), 1.48 (1H), 1.44 (1H), 1.40 (1H), 1.35 (1H), 1.28 (1H), 1.25 (1H), 1.10 (3H, s), 1.09 (1H), 1.02 (1H), 0.93 (3H, s), 0.93 (1H), 0.92 (1H), 0.92 (1H), 0.88 (3H, s), 0.86 (3H, s), 0.85 (3H, s), 0.80 (3H, s), 0.73 (3H, s). ^13^C-NMR (DMSO-*d*6, 150 MHz data from HSQC and HMBC) δ in ppm 179.6, 166.2, 159.5, 145.1, 142.5, 132.5, 125.0, 120.4, 115.9, 115.0, 83.6, 64.7, 54.4, 47.5, 47.0, 46.0, 45.3, 41.5, 41.2, 39.1, 38.9, 37.8, 33.7, 33.1, 32.5, 32.2, 30.5, 28.5, 27.3, 25.6, 23.5, 23.0, 23.0, 18.0, 17.7, 17.1, 16.2. Data were consistent with published values [52,53].

*trans*-*p*-coumaroyloxy maslinic acid (**9**) ^1^H-NMR (DMSO-*d*6, 600 MHz) δ in ppm 7.51 (1H, d, *J* = 16.0), 7.49 (2H, d, *J* = 8.7), 6.79 (2H, d, *J* = 8.7), 6.35 (1H, d, *J* = 16.0), 5.15 (1H, brt, *J* = 3.4), 4.50 (1H, d, *J* = 9.8), 3.68 (1H, brtd, *J* = 10.5, 4.4), 2.80 (1H dd, *J* = 13.7, 4.4),1.85 (1H), 1.85 (2H), 1.84 (1H), 1.72 (1H), 1.61 (1H), 1.60 (1H), 1.59 (1H), 1.49 (1H), 1.48 (1H), 1.45 (1H), 1.40 (1H), 1.36 (1H), 1.30 (1H), 1.25 (1H), 1.11 (1H), 1.11 (3H, s), 1.05 (1H), 0.96 (3H, s), 0.96 (1H), 0.93 (1H), 0.92 (1H), 0.88 (3H, s), 0.87 (3H, s), 0.85 (3H, s), 0.80 (3H, s), 0.73 (3H, s). ^13^C-NMR (DMSO-*d*6, 150 MHz data from HSQC and HMBC) δ in ppm 179.1, 166.7, 160.5, 144.7, 144.1, 130.0, 124.8, 120.9, 114.7, 116.0, 83.7, 64.9, 54.6, 47.4, 47.2, 46.0, 45.5, 41.4, 41.1, 39.1, 39.2, 37.6, 33.6, 33.0, 32.3, 32.4, 30.5, 28.6, 27.2, 25.6, 23.5, 23.0, 23.0, 18.0, 17.8, 17.1, 16.3. Data were consistent with published values [52].

*cis*-*p*-coumaroyloxy corosolic acid (**10**) ^1^H-NMR (DMSO-*d*6, 600 MHz) δ in ppm 7.66 (2H, d, *J* = 8.8), 6.82 (1H, d, *J* = 12.8), 6.72 (2H, d, *J* = 8.8), 5.79 (1H, d, *J* = 12.8), 5.08 (1H, brt, *J* = 3.5), 4.47 (1H, d, *J* = 9.9), 3.66 (1H, m), 2.17 (1H, dd, 10.8, 5.7),1.88 (1H), 1.87 (2H), 1.83 (1H), 1.92 (1H), 1.52 (2H), 1.54 (1H), 1.52 (1H), 1.47 (1H), 1.34 (1H), 1.38 (2H), 1.27 (1H), 1.26 (2H), 1.10 (3H, s), 0.95 (3H, s), 0.93 (1H), 0.93 (1H), 0.92 (1H), 0.90 (1H), 0.88 (3H, s), 0.86 (3H, s), 0.85 (3H, s), 0.80 (3H, s), 0.73 (3H, s). ^13^C-NMR (DMSO-*d*6, 150 MHz data from HSQC and HMBC) δ in ppm 179.2, 166.0, 159.1, 142.5, 139.4, 132.6, 125.3, 123.6, 116.2, 114.9, 83.5, 64.8, 54.4, 52.8, 47.7, 47.0, 46.7, 41.9, 39.3, 39.1, 38.8, 38.6, 37.8, 36.8, 32.7, 30.9, 28.5, 27.6, 24.1, 23.3, 23.0, 21.3, 17.9, 17.9, 17.7, 17.2, 16.5. Data was compared to published values [53].

*trans*-*p*-coumaroyloxy corosolic acid (**11**) ^1^H-NMR (DMSO-*d*6, 600 MHz) δ in ppm 7.51 (1H, d, *J* = 16.0), 7.50 (2H, d, *J* = 8.7), 6.78 (2H, d, *J* = 8.7), 6.34 (1H, d, *J* = 16.0), 5.18 (1H, brt, *J* = 3.5), 4.50 (1H, d, *J* = 9.9), 3.68 (1H, m), 2.17 (1H, dd, 10.8, 5.7),1.88 (1H), 1.87 (2H), 1.83 (1H), 1.92 (1H), 1.52 (2H), 1.54 (1H), 1.52 (1H), 1.47 (1H), 1.34 (1H), 1.38 (2H), 1.27 (1H), 1.26 (2H), 1.10 (3H, s), 0.98 (3H, s), 0.93 (1H), 0.93 (1H), 0.92 (1H), 0.90 (1H), 0.88 (3H, s), 0.86 (3H, s), 0.85 (3H, s), 0.80 (3H, s), 0.73 (3H, s). ^13^C-NMR (DMSO-*d*6, 150 MHz data from HSQC and HMBC) δ in ppm 179.2, 166.6, 160.7, 140.2, 139.4, 130.1, 124.7, 123.6, 114.6, 116.0, 83.5, 64.8, 54.4, 52.8, 47.7, 47.0, 46.7, 41.9, 39.3, 39.1, 38.8, 38.6, 37.8, 36.8, 32.7, 30.9, 28.5, 27.6, 24.1, 23.3, 23.0, 21.3, 17.9, 17.9, 17.7, 17.2, 16.5. Data was compared to published values [54].

### 3.3. UHPLC-MS/MS Analysis

The chemical analyses were performed on a UHPLC system (Dionex Ultimate 3000, Thermo Scientific^®^ equipped with a Photo Diode Array detector: 254, 280, 340 and 450 nm) coupled to a High-Resolution Mass Spectrometer (HRMS QqToF Impact II equipped with an electrospray ionization source, Bruker Daltonics, Bremen, Germany). The solutions were prepared by solubilizing 1 mg of dry extracts/fractions in 1 mL of methanol then filtered with a 0.2 μm PTFE syringe filter (Restek^®^, Lisses, France). The separations were carried out on an Acclaim RSLC C18 column (2.1 mm × 150 mm, 2.2 μm, Thermo Scientific^®^) at 40 °C by injecting 1 μL of the prepared solution. A smaller volume (0.25 μL and 0.5 μL) were injected when solutions were too concentrated. Additionally, some solutions were diluted 50 times to avoid detector saturation. The final ion intensities were normalized according to dilution factor.

Two chromatographic analytical methods, depending on the polarity of the extracts, were developed to obtain the best chromatographic separation, with H_2_O + 0.1% formic acid (solvent A) and acetonitrile + 0.1% formic acid (solvent B). The first program (pg 1) was as described: 2 min at 5% B, then 7 min ranging from 5 to 50% B followed by 2 min at 50% in isocratic mode. Finally, a 2 min isocratic wash at 100% B and a re-equilibration step at 5% during 3 min ended the analytical program (flow rate at 0.5 mL·min^−1^). It was performed on extracts BEAE, BF3, BF4, CEAE, CEWE, CF3, CF4, CF5, FEAE, FF2, FF3 and FF4. The second program (pg 2), used for FF1 that was the less polar extract, consisted of 2 min at 5% B followed by a linear gradient up to 100% B in 8 min, then 100% B for 3 min ended by a 3 min re-equilibration at 5% B (same flow rate). The injection of a formate acetate solution in basic media forming clusters on the studied mass range was used for mass calibration before each analysis. Mass spectra were acquired in DDA-MS^2^ negative mode (40 eV; 3 main precursors) ranging for 50 to 1200 *m*/*z* at 2 Hz. The nebulizer pressure was set at 50.8 psi, the capillary voltage at 3000 V, the dry gas flow rate at 12 L·min^−1^, and dry temperature at 200 °C.

### 3.4. Construction of the Molecular Network

The raw MS^2^ data files were converted to mzXML via a Bruker script run on Compass Data Analysis version 5.0 (Bruker Daltonik, Bremen, Germany). The MZmine version 2.53 was then used for pre-processing. The files were filtered to remove the 0.00–0.37 min calibration portion of each analysis. A centroid mass detection of MS^1^ at a noise level of 1.0E2 and MS^2^ noise level of 1.0E2, generated a mass list. The *m*/*z* tolerance was set to 0.01 Da. The feature list was used to build chromatograms using ADAP. The minimum group number of scans was set at 3, the group intensity threshold was set at 3000 and the minimum highest intensity was set at 4000. Deconvolution of the feature lists was done with the wavelets (ADAP) algorithm. The feature lists were deisotoped and the features of the thirteen samples were aligned. Finally, duplicates were filtered, whereas fragments and adducts were searched. The resulting aligned list of features was gap-filled (intensity tolerance = 10%, *m*/*z* difference = 0.002, rt difference = 0.2) then exported in a mgf file along with their feature quantification table in csv format. The feature quantification table values were uploaded onto the FBMN page of GNPS version 1.3.11 [55]. The precursor and fragment ion masses were both set to 0.02 Da. The molecular network was visualized on Cytoscape version 3.7.2. Analyses of all extracts were used for the construction of FBMN, except BF1, BF2, CF1 and CF2 for which the bioactivity was found to be null.

### 3.5. Proliferation Assay of Hair Follicle Dermal Papilla Cells

The cell proliferation protocol used was described in previous publications [7,8]. Human hair follicle dermal papilla cells were purchased from Promocell (Heidelberg, Germany) and grown in follicle dermal papilla cell growth medium (Promocell, Sickingenstr, Heidelberg, Germany) supplemented with 100 U·mL^−1^ penicillin and 100 μg·mL^−1^ streptomycin (Gibco). The cells were cultured in a humidified atmosphere at 37 °C and 5% CO_2_. The Cell Proliferation kit I MTT assay (Sigma Aldrich^®^, St. Louis, MI, USA) was performed to determine the cell proliferation activity of the extracts and fractions. Cells were seeded at 10^4^ for 24 h into 96-well plates. They were treated with 200 μL of increasing concentrations of the extracts, ranging from 0.1 μg·mL^−1^ to 50 μg·mL^−1^. The control group consisted of DMSO diluted in medium to concentrations ranging from 0.001% to 0.5%, similarly to those of the extracts. The plates were incubated at 37 °C, 5% CO_2_ for 24h. The supernatant was then discarded and 100 μL of 10% MTT in fresh medium was added to the wells and incubated for 4 h before adding 100 μL of solubilization solution. The plates were incubated overnight, and the absorbance was read on a Multiskan GO spectrophotometer (Thermoscientific, Inc., Waltham, MA, USA) at 570 and 690 nm. All tests were done at least in triplicates. The absorbance of each extract concentration was normalized to its corresponding control. The cell proliferation values for 50 μg·mL^−1^ were used here.

### 3.6. Statistical Analyses

A PCA-X analysis was conducted on SIMCA^®^ version 17 (Umeå, Sweden) on MS^1^ quantitative data of the precursors from MS^2^ analysis. Data were log-transformed, and center-scaled.

The Pearson correlation coefficient between feature intensities and biological activities of the thirteen extracts and fractions was based on the protocol proposed by Nothias et al., (2018) using a *R* script (https://github.com/DorresteinLaboratory/Bioactive_Molecular_Networks/blob/master/Bioactive_Molecular_Networks_v1.1_MZmine2.r, accessed on 11 February 2021).

## 4. Conclusions

The Feature Based Molecular Network paired with the cell proliferation bioactivity enabled us to deepen our knowledge of bioactive metabolites found in plants traditionally used for hair care in French Polynesia. We were able to annotate iridoids such as swertiamarin and loganic acid for the first time in the fruits of *F. berteroana* as well as discover other metabolites like C-glycosylated and O-glycosylated flavonoids that are less common in the *Fagraea* genus and link them to dermal papilla cell proliferation. No previous research had identified a coumarin and triterpenoids from *F. berteroana* as reported here. Furthermore, the constituents of *B. pilosa* and *C. inophyllum* were linked for the first time to this bioactivity. Their mode of action is likely mediated via the activation of the Wnt/β-catenin pathway according to our previous findings.

To our knowledge, this study describes the first application of Bioactivity Based Molecular Networks on hair related activities. It shows that this method could be a great tool for prospective work on the traditional uses of plants in French Polynesia, or in other regions.

Our findings emphasize the diversity of metabolites in our plant extracts and highlight the valorization potential of each plant species regarding its bioactive compounds. Furthermore, the association between diabetes and hair loss could open an avenue for potential cross-investigations between the two research fields.

## Figures and Tables

**Table 1:** LC-MS/MS data of features detected in extracts and fractions (“+” and “−“ as positive correlation and negative correlation to cell proliferation respectively).

Exp. *m/z*	Ion Type	Molecular Formula (Δppm)	Annotation	Comparison Source	Correlation to Cell Proliferation
111.0088	[M − H]^−^	C_5_H_4_O_3_ (0.3)			+
163.0401	[M − H]^−^	C_9_H_8_O_3_ (0.2)	*p*-coumaric acid	Massbank:RP016813	+
173.0456	[M − H]^−^	C_7_H_10_O_5_ (0.3)	shikimic acid	Massbank: RP017513	+
339.0722	[M − H]^−^	C_15_H_16_O_9_ (0.3)			+
353.0878	[M − H]^−^	C_16_H_18_O_9_ (0.0)	chlorogenic acid	MoNA:VF-NPL-QEHF011308	+
375.1301	[M − H]^−^	C_16_H_24_O_10_ (1.1)	loganic acid	MoNA:VF-NPL-QTOF002452	+
419.1197	[M + HCOO]^−^	C_17_H_24_O_12_ (0.5)	swertiamarin	Massbank:PR307657	+
431.0981	[M − H]^−^	C_21_H_20_O_10_ (−0.6)	isovitexin	Massbank:PR307135	+
431.0982	[M − H]^−^	C_21_H_20_O_10_ (−0.4)	afzelin	MoNA:VF-NPL-QEHF015100	−
447.0932	[M − H]^−^	C_21_H_20_O_11_ (−0.2)	luteolin-8-C glucoside	Massbank:PR305779	+
447.0933	[M − H]^−^	C_21_H_20_O_11_ (0.0)	luteolin-7-O-glucoside	Massbank:PR305563	+
447.0934	[M − H]^−^	C_21_H_20_O_11_ (0.3)	quercitrin	Massbank:FIO00585	−
463.0884	[M − H]^−^	C_21_H_20_O_12_ (0.4)	isoquercitrin	Massbank:FIO00167	+
477.0671	[M − H]^−^	C_21_H_18_O_13_ (−0.8)	quercetin 3-O glucuronide	MoNA:VF-NPL-QEHF015166	−
511.0548	[M − H]^−^	C_21_H_20_O_13_S (−0.8)	kaempferol-C_6_H_10_O_7_S	[7]	+
515.1769	[M − H]^−^	C_23_H_32_O_13_ (−0.2)			−
527.0501	[M − H]^−^	C_21_H_20_O_14_S (0.0)	quercetin-C_6_H_10_O_7_S	[7]	+
575.1195	[M − H]^−^	C_30_H_24_O_12_ (0.0)	procyanidin A1		+
577.1338	[M − H]^−^	C_30_H_26_O_12_ (−2.3)	procyanidin B2	MassBank: BS003942	+
577.1346	[M − H]^−^	C_30_H_26_O_12_ (−1.0)	procyanidin B1	MassBank:BS003943	+
609.1465	[M − H]^−^	C_27_H_30_O_16_ (0.6)	rutin	Massbank:FIO00596	+
623.1612	[M − H]^−^	C_28_H_32_O_16_ (−0.9)	isorhamnetin-3-O-rutinoside	Massbank:PR305498	+
865.2005	[M − H]^−^	C_45_H_38_O_18_ (2.3)	procyanidin C1	MassBank: PR101005	+

## Data Availability

The GNPS generated LC-MS/MS network can be accessed at: https://gnps.ucsd.edu/ProteoSAFe/status.jsp?task=b62e4e309c354b1abafdb14657e6d093, accessed on 20 December 2021.

## Supplementary Materials

The following are available online, Table S1: Cell proliferation values before and after ln transformation as prepared for the feature quantification table, Table S2: List of significant features according to Pearson correlation coefficient for model including all three species.

## Sample Availability

Samples of the compounds are available in very limited quantity from authors.
