# Supplementary material for: Feature-Based Molecular Networks Identification of Bioactive Metabolites from Three Plants of the Polynesian Cosmetopoeia Targeting the Dermal Papilla Cells of the Hair Cycle
Source: Molecules. 2021 Dec 24;27(1):105. doi: 10.3390/molecules27010105 (PMC8746341; doi:10.3390/molecules27010105)
Supplement: Supplementary file 1 [file molecules-27-00105-s001.zip › molecules-1463789-supplementary.pdf]

SUPPLEMENTARY MATERIAL

Feature-Based Molecular Networks Identification of Bioactive Metabolites from Three Plants of the Polynesian Cosmetopoeia Targeting the Dermal Papilla Cells of the Hair Cycle

Kristelle Hughes[1], Raimana Ho[1], Stéphane Greff[2], Gaëtan Herbette[3], Edith Filaire[4,5], Edwige Ranouille[4], Jean-Yves Berthon[4] & Phila Raharivelomanana[1]

[1] Université de la Polynésie Française, IFREMER, ILM, IRD, EIO UMR 241, BP 6570, F-98702 Faaa, Tahiti, French Polynesia; kristelle.hughes@doctorant.upf.pf (K.H.); raimana.ho@upf.pf (R.H.)
[2] Institut Méditerranéen de Biodiversité et d'Ecologie Marine et Continentale (IMBE), UMR 7263 CNRS, IRD, Aix Marseille Université, Avignon Université, Station Marine d'Endoume, rue de la Batterie des Lions, 13007 Marseille, France; stephane.greff@imbe.fr
[3] Aix Marseille Université, CNRS, Centrale Marseille, FSCM, Spectropole, Service 511, Campus Saint-Jérome, 13397 Marseille, France; gaetan.herbette@univ-amu.fr
[4] Greentech SA, Biopôle Clermont-Limagne, 63360 Saint-Beauzire, France; edithfilaire@greentech.fr (E.F.); developpement@greentech.fr (E.R.); jeanyvesberthon@greentech.fr (J.-Y.B.)
[5] Université Clermont Auvergne, UMR 1019 INRA-UcA, UNH (Human Nutrition Unity), ECREIN Team, 63000 Clermont-Ferrand, France
* Correspondence: phila.raharivelomanana@upf.pf

Table S1. Cell proliferation values before and after ln transformation as prepared for the feature quantification table

	BEAE	BF3	BF4	CEWE	CEAE	CF3	CF4	CF5	FEAE	FF1	FF2	FF3	FF4
Values at 50 $\mu g.ml^{-1}$	1.29	1.09	1.06	1.50	0.85	0.76	0.89	1.20	1.31	1.43	1.17	1.34	1.38
Ln-transformed values	0.2546	0.08618	0.05827	0.4055	-0.1625	-0.2744	-0.1165	0.1823	0.2700	0.3577	0.1570	0.2927	0.3221

Table S2. List of significant features according to Pearson correlation coefficient for model including all three species

m/z	retention time (min)	correlation	*p*-value
453.1552	11.81	-0.79	0.001
259.0243	10.98	-0.75	0.003
489.1044	9.25	-0.73	0.005
437.0881	11.61	-0.73	0.005
191.042	0.82	-0.71	0.006
463.0876	2.46	-0.71	0.007
575.2442	10.95	-0.71	0.007
607.2669	11.48	-0.70	0.008
563.272	9.12	-0.70	0.008
340.8268	15.42	-0.70	0.008
325.0437	13.50	-0.69	0.008
311.0926	12.73	-0.69	0.008
409.1657	11.50	-0.68	0.01
159.0664	7.19	-0.68	0.01
239.129	12.92	-0.65	0.02
433.2201	11.68	-0.64	0.02
303.051	9.23	-0.64	0.02
243.0299	10.49	-0.63	0.02
273.0405	12.25	-0.62	0.02
244.0649	1.47	-0.62	0.02
303.051	11.87	-0.61	0.03
503.1945	8.92	-0.60	0.03
662.2977	9.82	-0.59	0.03
160.8294	0.90	-0.59	0.04
663.138	5.78	-0.58	0.04
287.1976	8.60	-0.58	0.04
148.9409	14.91	-0.58	0.04
513.3066	11.74	-0.58	0.04
253.1234	8.94	-0.58	0.04
575.2477	11.22	-0.58	0.04
449.1542	11.47	-0.58	0.04
576.258	10.66	-0.58	0.04
248.9383	0.75	-0.57	0.04
335.0934	11.93	-0.57	0.04
223.1151	9.21	-0.57	0.04
303.051	10.18	-0.56	0.04
233.135	10.97	-0.56	0.05

293.1855	10.97	-0.56	0.05
116.9194	15.56	-0.56	0.05
195.1223	9.84	-0.56	0.05
194.0653	10.06	-0.56	0.05
293.1868	11.23	-0.56	0.05
115.9117	12.97	-0.56	0.05
539.0931	11.78	-0.56	0.05
303.0915	7.71	-0.56	0.05
341.0703	10.51	-0.55	0.05
265.108	10.16	-0.55	0.05
339.0722	5.36	0.55	0.05
425.1248	12.44	-0.55	0.05
559.2509	11.81	-0.55	0.05
111.0088	1.10	0.55	0.05
589.2222	9.40	-0.55	0.05
551.1621	6.03	-0.55	0.05
